# The Human Library and the development of cultural awareness and sensitivity in occupational therapy students: a mixed methods study

**DOI:** 10.3389/fmed.2023.1215464

**Published:** 2023-09-28

**Authors:** Kirsty Pope, Heather Hewlin-Vita, Eli Mang Yee Chu

**Affiliations:** Department of Occupational Therapy, Monash University, Melbourne, VIC, Australia

**Keywords:** cultural competency, Human Library, health professions education, education research, occupational therapy

## Abstract

**Introduction:**

Monash university in Australia has adopted the Human Library as a teaching activity aimed at enhancing occupational therapy (OT) students’ cultural awareness and sensitivity; however, its effect on cultural competence in healthcare profession students has not been previously investigated.

**Aim:**

To examine whether participation in a Human Library can facilitate the development of OT students’ cultural awareness and sensitivity and to understand the factors contributing to changes in cultural competence from the students’ perspective.

**Method:**

This is a mixed-method study. Quantitative data was collected by participant characteristic survey and pre/post-Human Library cultural competence assessment (CCA). The quantitative data was used to inform the selection of participants and questions for the second qualitative phase. Quantitative data were analyzed using independent samples and paired *t*-tests, analysis of variance and Pearson correlation. Qualitative data were analyzed using coding reliability thematic analysis.

**Results:**

Statistically significant increases were noted in CAS, CCB and overall CCA scores from pre- to post-Human Library. Participant characteristics such as gender, work experience in aged care and the health sector had a positive effect on pre-Human Library CCA results. Qualitative data demonstrated that participants perceived they had a level of cultural competence prior to attending the Human Library however, still made gains in cultural awareness and sensitivity and were motivated and inspired to provide culturally congruent healthcare.

**Conclusion:**

The Human Library can be considered for inclusion within a suite of learning methods for healthcare professional student curricula as a cost-effective, flexible teaching method for the development of competencies for culturally congruent healthcare.

## Introduction

In 2020, 7.6 million migrants lived in Australia representing 29.8% of the population (1). According to the United Nations (2020) this puts Australia within the top ten countries around the world with the highest number of foreign-born residents (2). Healthcare professionals do not always possess the skills required to service diverse populations and systemic factors can impact on users’ ability to navigate and utilize healthcare, resulting in health care disparity (3). Frameworks to help address this disparity have been proposed within healthcare, including cultural awareness, cultural sensitivity, cultural competence and cultural safety (4). All of these frameworks require health professionals to reflect on their own culture so they are in a better position to understand the culture of those they work with (5). Given the initiative to build methods of teaching culturally congruent care into occupational therapy curriculum/practice (6) further rigorous investigation is warranted due to a lack of evidence on the best pedagogical methods to achieve this.

The authors of this article are academics and occupational therapists who aimed to enhance teaching and learning on cultural competency and culturally safe practice in the curricula of undergraduate (UG) and master-entry level occupational therapy (MOTPrac) programs provided by a university in Victoria, Australia. Prior to 2018, the curricula focused on understanding culture as one of the contextual determinants of health, fostering cultural awareness and knowledge. Teaching was delivered mainly through lectures, seminars, tutorials, and online modules for most students. A small number of UG students were able to participate in inter-cultural experiences overseas. In 2018 the revised Australian Occupational Therapy Competency Standards (7) placed more emphasis on the requirement for OTs to practice in a culturally safe and sensitive manner. In addition to learning within the university setting, students were expected to develop and practice cultural competencies in their professional practice education fieldwork placements. In order to better prepare our students for culturally safe and sensitive practice, we explored adapting and integrating the Human Library program, to create an educational experience to encourage students to interact with people from diverse backgrounds and to have deep reflection on their cultural competencies and learning.

The Human Library was a social movement, started in 2000 in Copenhagen, aimed to provide a positive framework for conversations, which challenge stereotypes and prejudices and therefore, build positive relationships between people and promote inclusion (8). The Human Library is similar to a standard library except that the books are people with a story and the reading is a conversation (9). It unites individuals who may not otherwise interact with each other (8) and, as such, can be considered a social experiential learning opportunity. Although positive effects of a Human Library are reported for improving mental health literacy (10); social inclusion and promoting recovery (11); respect and reducing bias (12); reframing attitudes (13); and reducing prejudice (14) its effect on cultural competence in student healthcare professionals has not been investigated.

This research was guided by the cultural competence model (15, 16) which conceptualizes cultural competence, as a non-linear, interconnected, three-dimensional jigsaw, and ever-evolving process towards culturally congruent care. The model has three layers, each containing multiple elements (Table 1). The model values diverse cultural experiences and posits that recgonising differences *and* similarities between cultures allows personal insight and space for cultural sensitivity to emerge (15). What follows are culturally competent behaviors such as the ability to adapt and negotiate for better health outcomes (15). This model was chosen because it is well-cited in healthcare literature (16–22) and provides a framework for analyzing the development of cultural competencies of students after attending a Human Library embedded in a teaching unit.

**Table 1:** Elements of each layer of the 3D model Schim and Doorenbos (16).

Provider layer (the healthcare professional or student)	The person moves between the following stages towards culturally competent behavior: cultural diversity, awareness, sensitivity, and culturally competent behaviors
Client layer	Individual, family, group, or whole community who are the focus of healthcare services Composed of their attitudes, beliefs and behaviors that produces similarities or differences between individuals, cultural groups, and subgroups
Outcome culturally congruent care	Outcome is achieved as providers mindfully interact with culturally diverse clients and the provider and client layers unite

The Research questions for the study were:

1. What is the effect of participation in a Human Library on the development of cultural awareness and sensitivity in undergraduate OT students?
2. What is the students’ perspective on the influence of the Human Library on their cultural competence?

## Methods

We chose a mixed methods explanatory sequential pre-post design without a comparison group, to explore the effectiveness, and student’s perception of, a Human Library program in developing cultural competency. We recognized that a combination of quantitative and qualitative data results in a more comprehensive understanding of a topic than either method alone (23). The quantitative data analysis was used to refine the second qualitative phase including, participant selection, and focus groups questions. In turn, the qualitative data was used to explain and interpret quantitative results (23).

### Educational intervention

The design of the Human Library program embedded in the curriculum is based on experiential learning theory and principles to provide opportunities for students to interact with people from diverse ethnic, age, social backgrounds as well as people or carers with lived experience of health issues. Students are required to complete an online module introducing cultural concepts and to complete a self-reflection task before attending a Human Library session. The pre-Human Library reflection tasks ask students to reflect on their own culture, how their culture influenced them, their perception of cultural competency and any preconceived ideas of two human books selected from the “books” available to them in the Human Library session. Students attend a Human Library session, after which they complete a reflection on the experience, noting any challenges to their preconceived ideas and how they could apply what they learned in future OT practice. Prior to COVID 19 travel restrictions the Human Library took place face to face on the university campus. In April 2021, 98 onshore and offshore OT students enrolled in an undergraduate 2nd year unit took part in the Human Library sessions via video conferencing.

Fifteen volunteers from the university staff network and from the community were recruited to be human books. Academic staff organizing the Human Library explained the purpose of the Human Library to volunteers, and discussed the guidelines about protecting privacy and emotional risk when books share their stories. Students were briefed about the expectation to be respectful, demonstrate professional behavior, communication and to protect the privacy of the books, which are essential competency for health professionals. Two Human Library sessions were run online via a video conferencing platform, each with 15 human books, and 48 and 50 students, respectively. Groups of three to five students engaged in conversation for 25 min each time with two different human books. The human books told a personal story followed by questions from, and discussion with, the students.

### Recruitment

Second year undergraduate occupational therapy students (*n* = 98) enrolled in a teaching unit in which the Human Library program is embedded were invited to participate in the study through an announcement on the online learning management system. Due to a small single sample pool, quantitative sampling involved non-probabilistic convenience sampling. Students were invited to participate in the study by completing pre and post Human Library surveys and attending a focus group. Explanatory statements and consent forms were sent to eligible students via email approximately 2 weeks prior to the Human Library. Potential participants were invited to attend one of two online recruitment sessions, where information about the study were presented, and opportunities for questions provided. All the recruitment procedures were administered by a member of the research team not directly involved in teaching these undergraduate students (KP) to avoid conflict of interest and coercion.

To ensure participants with a range of characteristics and experience were included, maximum variation sampling (24) was used to group participants who provided informed consented to participate in a focus group according to their gender, age, ethnicity, location (onshore or overseas), work experience, cultural learning experience, and exposure to people from different cultures.

### Data collection

A Qualtrics survey was used to gather data on participant characteristics (age, gender, onshore/offshore, home country and number of languages spoken) and past experience in cultural competency training.

The cultural competence assessment (CCA) (25), a 25-item self-report tool was used to measure cultural competency across two factors: cultural awareness and sensitivity (CAS), and culturally competent behaviors (CCB). The CCA, based on the cultural competence model (15, 16) was chosen as it is quick to administer and suitable for participants with varying levels of education from less than high school to graduate school level (19, 25). The CCA is recommended for measuring cultural competence in nurses (20) and is suitable to use with other healthcare providers (including clerical, administrative staff, hospice workers, therapists, nursing assistants and social workers) (19). The CCA uses a seven-point Likert scale. The CAS subscale consists of 11 items (7 = strongly agree, 1 = strongly disagree), with a maximum scale score of 77; and CCB subscale consists of 14 items (7 = always, 1 = never), with a maximum subscale score of 98 (19). Respondents are given choices to answer each question with the answer “no opinion” or “not sure,” a score four out of seven, equivalent to the score of “natural” was allocated to these responses.

The CCA is sensitive to educational methods targeting cultural competence making it suitable to use in research (19, 20, 25) and has good internal consistency (Cronbach’s *α* 0.86–0.93) (20) indicating that items proposing to measure the same construct produce similar scores (26). It also has good test-retest reliability when tested on 51 hospice workers of varying disciplines and education levels (*r* = 0.85, *p* = 0.002) (19) and has sufficient content validity (19, 20). Construct validity has been assessed with 25 items having factor analysis loadings above 0.42 (19).

**Figure 1:**
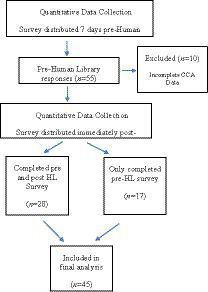
Quantitative data collection.

Data were collected at three time points. Participants completed the pre-Human Library CCA survey 7 days prior to the Human Library event. Immediately following the Human Library, participants completed the post-Human Library CCA survey (Figure 1).

After students had received the results for the academic unit, consenting participants attended a 60 min online semi-structured audio-recorded focus group to share their views of the Human Library. Focus group questions were informed by the quantitative survey data and designed to facilitate discussion of the participants’ prior level of cultural awareness and sensitivity, their perspective of the Human Library experience, levels of self-efficacy for cross-cultural interactions, the impact of COVID-19 on opportunities to interact with people from different backgrounds, and what participants would do differently as a result of the Human Library. Quantitative data analysis resulted in the addition of two questions to clarify unexpected quantitative results, firstly, the lack of significant change in pre-post cultural awareness and sensitivity and to investigate the effect of COVID-related restrictions on participants’ intercultural interactions.

### Quantitative analysis

All data were cleaned and de-identified prior data analysis using Statistical Package for the Social Sciences (SPSS) (version 27.0). Participant characteristic data were summarized using descriptive statistics. Total CCA scores and that of each factor (CAS and CCB) and each CCA item were assessed for normality by analyzing the distribution of frequencies (26). Paired *t*-tests were used to evaluate differences between pre-/post-CCA scores for the total scores, CAS and CCB subscale scores. *t*-test results were considered to be significant if *p* ≤ 0.05 (26). Effect sizes, using Cohen’s *d*, were calculated to assess the magnitude of the differences of CCA, CAS, and CCB score pre and post participation in the Human Library (26).

Independent *t*-test was used in subgroup analysis to compare CCA, CAS and CCB scores for the following factors: participants’ location at the time of the study (onshore or offshore). with or without previous work experiences in aged care, disability and health sector, with or without previous cultural learning experiences, and those who completed both pre and post Human Library surveys or not. In the survey, we asked participants whether they had previous work experiences in a variety of paid or non paid work in the aged care, disability and health sector. We grouped the responses of all types of aged care, disability and health work experiences into one group “with previous work experiences in aged care, disability and health” to compare with participants who did not have work experiences in these fields. One-way analysis of variance (ANOVA) was to be used for subgroup analysis to compare CCA, CAS and CCB scores for the following factors: age groups gender (male, female, prefer not to say), and, frequency of intercultural interactions at work/university and home/leisure time. The results of the quantitative data analysis were used to refine the second qualitative phase of the research including, participant selection and refinement of interview questions (23).

### Qualitative analysis

The aim of qualitative analysis was to provide a rich description of the data set on a new area of research (27) as is the case with the Human Library. Each researcher first engaged in a process of reflexivity where their own unconscious bias was explored as an integral component of the qualitative phase of the research (27). The qualitative analysis followed Braun and Clarke’s (27) six-phase thematic analysis, incorporating the steps recommended by Liamputtong (24), within step 1: familiarization of data. This involved each author reading and re-reading the transcripts to develop familiarity with the content and to gain an understanding of what participants as a group were saying (24). Through continued examination of the transcripts, the authors were able to move to steps two and three with the generation of codes, a codebook (27) and each author reviewing the codes to define and name themes (24, 27). Step four involved the comparison of themes, and step five, reaching a consensus on themes by discussion. The completion of steps 1–5 resulted in unbiased themes ready for reporting. Qualitative results were compared with quantitative results through methodological triangulation and as the final stage; multiple quotations demonstrating the themes were chosen to be presented in text (24, 28)to ensure data triangulation (24).

### Ethical approval and considerations

Approval was granted by the university’s Human Research Ethics Committee (27411) and considered low risk. To avoid potential coercion, the researcher involved with teaching and coordination of the undergraduate curriculum was not involved in recruitment. To avoid breaches of confidentiality, the researchers involved with teaching and coordination of the undergraduate (EC) and masters (KP) curricula did not have access to identifiable data and the UG coordinator (EC) was not involved in interviewing students in phase two. Quantitative and qualitative data were de-identified by a researcher not involved in teaching OT students (HH-V) and stored in a separate password protected file, accessible by all researchers for analysis.

## Results

### Participant characteristics

Fifty-five out 98 students participated in the study, the response rate was 57%. Ten responses were excluded due to incomplete CCA data occurring when participants started responding to demographic questions but stopped before completing the CCA, resulting in a final sample size of 45 (male = 12, female = 32, one prefers not to say). Twenty-eight participants (male = 8, female = 20) answered both the pre and post-Human Library surveys, with 17 participants completing the pre-Human Library only.

The majority of participants were aged 18–24 years, more than half of the participants were from North-East Asia including China, Hong Kong, and Macau. Approximately one third were from Australia, and the remaining were from Afghanistan, Brunei, England, France, India, or Saudi Arabia. There were 39 participants studying in Australia at the time of the research, with six offshore. Whilst the majority of participants did not report their work experience, those that did, noted the areas of aged care, health and/or disability.

Many participants reported infrequent intercultural interactions with 40% having weekly or less than weekly intercultural interactions at work, volunteering and/or university and 80% having weekly or less than weekly intercultural interactions at home or during leisure/social activities. The majority of participants (73%) reported speaking two or more languages and 96% of participants indicated interest in learning about different cultures and building intercultural skills. Detailed demographic information of the participants are presented in Tables 2, 3.

**Table 2:** Characteristics of participants at baseline—age, gender, location and work experience.

Participants characteristics	All students at baseline (*n* = 45)				Students who completed both pre and post Human Library survey (*n* = 28)				
	*N* (%)	CAS scores *M* (SD)	CCB scores *M* (SD)	CCA scores *M* (SD)	*N* (%)		CAS scores *M* (SD)	CCB scores *M* (SD)	CCA scores *M* (SD)
*Age*									
18–24	42 (93%)	61.95 (7.26)	59.29 (10.69)	121.24 (13.93)	26 (93%)	Pre	63.88 (5.81)	60.54 (9.34)	124.42(9.24)
						*Post*	*65.57 (6.29)*	*66.81 (12.03)*	*132.38 (12.88)*
25–34	2 (4%)	61.50 (3.54)	41.50 (2.12)	103.00 (5.66)	1 (3.5%)	Pre	59.0000	40.00	99.00
						*Post*	*64.00*	*42.00*	*106.00*
35–44	1 (2%)	73.00	57.00	130.00	1 (3.5%)	Pre	73.00	57.00	130.00
						*Post*	*73.00*	*74.00*	*147.00*
*Gender*									
Male	12 (27%)	58.92 (5.76)^b^	58.17 (10.32)	117.08 (11.98)	8 (29%)	Pre	59.25 (5.80)^c^	60.63 (11.24)	119.88 (13.42)
						*Post*	*63.50 (7.84)*	*66.00 (13.10)*	*129.50 (16.22)*
Female	32 (71%)	64.16 (5.93)^b^	59.25 (10.77)	123.41 (12.14)	20 (71%)	Pre	65.95 (4.91)^c^	59.30 (9.47)	125.25 (8.50)
						*Post*	*66.70 (5.42)*	*66.25 (12.71)*	*132.95 (12.87)*
Prefer not to say	1 (2%)	38.00	36.00	74.00	0 (0%)		—	—	—
*Student location*									
Onshore	39 (87%)	62.28 (7.57)	57.83 (10.97)	120.12 (14.37)	26 (93%)	Pre	63.96 (6.06)	59.62 (10.02)	123.58 (10.55)
						*Post*	*66.31 (5.83)*	*66.00 (12.96)*	*132.31 (14.15)*
Offshore	6 (13%)	61.50 (4.72)	62.50 (11.00)	124.00 (12.62)	2 (7%)	Pre	65.00 (5.66)	60.50 (9.19)	125.50 (3.54)
						*Post*	*59.00 (9.90)*	*68.50 (7.78)*	*127.50 (2.12)*
Previous work experiences in aged care, disability and healthcare^a^	14 (31%)	65.43 (5.46)^d^	59.64 (10.49)	125.07 (11.80)	10 (26%)	Pre	67.00 (4.67)^e^	61.00 (11.50)	128.00 (12.08)
						*Post*	*68.10 (6.77)*	*63.40 (15.17)*	*131.50 (15.60)*
No work experience in these fields	31 (69%)	60.71 (7.51)^d^	57.90 (11.31)	118.61 (14.73)	18 (64%)	Pre	62.39 (6.03)^e^	58.94 (9.01)	121.33 (8.40)
						*Post*	*64.50 (5.69)*	*67.72 (11.07)*	*132.22 (12.97)*

a Frequency of work experience: unpaid caregiver (*n =* 5), health professional assistant (*n =* 4), personal/community/disability support worker (*n =* 2), shadowing a health professional (*n =* 1), aged or community care volunteer (*n =* 1), others (*n =* 1). One participant had work experience as an unpaid caregiver and healthcare professional assistant.

b Significant difference observed in baseline CAS score between male and female students, *t*(42) = −2.630, *p* = 0.012, response “prefer not to say” was not included in the analysis.

c Significant differences observed between male and female CAS score (students completed pre and post test), *t*(26) −3.099, *p* = 0.005.

d Significant differences in baseline CAS scores observed between students with/without work experience in aged care, disability or health sector, *t*(43) 2.108, *p* = 0.041.

e Significant differences observed between students (completed pre and post test) with/without work experience in aged care, disability or health, CAS score, *t*(26) 2.089, *p* = 0.047.

**Table 3:** Participants ethnicity and country of origin at baseline (*n =* 45).

Ethnicity/count	Country of origin										
	India	Hong Kong	Australia	Macau	England	France	China	Afghanistan	Saudi Arabia	Brunei	Total
Aboriginal and/or Torres Islander			1								1
Australian			10		1						11
North-West European						1					1
Southern and Eastern European			1								1
North African and Middle Eastern									1		1
South-East Asian	1	11	2	2			1			1	18
North-East Asian		3					1				4
South and Central Asian	1	2					1	1			5
Prefer not to say		3									3
Total	2	19	14	2	1	1	3	1	1	1	45

Thirty participants reported prior cultural learning experiences such as courses/workshops and living/studying abroad, whilst the remaining five did not (see Table 4).

**Table 4:** Summary of number of participants participating in previous cultural learning experiences (*n =* 45).

Cultural learning experiences	Number of students
Cultural learning course only	5
Cultural learning workshop only	11
Lived abroad only	3
Studied abroad only	6
Cultural learning immersion program only	1
International homestay only	1
Other cultural learning experience only	1
Multiple cultural learning experiences	12
Not participated in any cultural learning experience	5

### Quantitative analysis

#### Cultural competency as measured by the CCA

##### Cultural awareness and sensitivity

There was a significant increase in CAS mean scores (*p* = 0.043) from the pre-Human Library experience (*M* = 64.04, SD = 5.93, range 50–73), to post-Human Library experience (*M* = 65.79SD = 6.23, range 49–75) out of 77. The mean increase in the CAS scores was 1.75, 95% CI (− 0.27, −3.77) with a small effect size (Cohen’s *d* = 0.336).

##### Culturally competent behaviors

There was a statistically significant increase in CCB scores (*p* = 0.004) from pre-Human Library (*M* = 59.68, SD = 9.81, range 40–82) to post-Human Library (*M* = 66.18, SD = 12.58, range 42–97) out of 98. The mean increase in CCB scores was 6.50, 95% CI (1.86, 11.1) with a moderate effect size (Cohen’s *d* = 0.544) indicating that the Human Library had a moderate positive influence on self-perceived culturally competent behaviors.

##### Cultural competence

There was a statistically significant increase in CCA scores (*p* = 0.001) from pre-Human Library (*M* = 123.71, SD = 10.18, range 99–145) to post-Human Library (*M* = 131.96, SD = 13.68, range 106–165) out of 175. The mean increase in CCA scores was 8.25, 95% CI (3.12 to 13.38) with a moderate effect size (Cohen’s *d* = 0.624) indicating that the Human Library had a moderate positive influence on overall self-perceived cultural competence.

#### The relationship between participant characteristics and CCA scores at baseline

##### Gender

Before attending the Human Library, female students had significantly higher CAS scores (*M* = 64.16, SD = 5.93) and CCA scores (*M* = 123.41, SD = 12.14) in comparison to male students CAS scores (*M* = 58.92, SD = 5.76) and CCA scores (*M* = 117.08, SD = 11.98); those prefer not to report their gender (CAS = 38, CCA = 74) based on analysis of variance. Only one participant selected the gender option of “prefer not to say” and the CAS and CCA scores for this participant were much lower than the mean scores of the male and female groups. We used the independent *t*-test to do further analysis to compare the male and female baseline CAS and CCA scores. There were significant differences in both baseline CAS scores between male and female, (*p* = 0.012). No significant differences were detected between males and females for pre-Human Library CCB and CCA scores. Age. Almost all the respondents were within the 18–24 years old age group (*n* = 42), two were between 25–34 years old age group and one was between 35 and 44 years old. We did not perform subgroup analysis for age as a factor due to the uneven distribution between the three different age groups.

##### Work experiences

Participants who have work experiences in aged care, disability and health have significantly higher pre-Human Library CAS, CCB and CCA scores compared to those who do not. However, only the differences of the CAS scores between the groups were significantly different (*p* = 0.041).

##### Previous cultural learning experiences/interactions

There were no significant differences detected in pre-Human Library CAS, CCB and CCA scores between participants with or without previous cultural learning experience that may include cultural learning courses or workshops, lived abroad, studied abroad, cultural immersion programs or international homestay experiences. We also found no significant associations between the pre-Human Library CAS, CCB, CAS scores and the frequency of participants engaged in intercultural interactions at work or university, at home or during leisure activities.

##### Geographical location

There were no significant differences in pre-Human Library CAS, CCB and CAS scores between participants who were onshore or offshore.

##### Completion of pre and post survey

There was a significant difference (*p* = 0.025) in pre-Human Library CAS scores for participants who completed pre and post CCA (*n* = 28, *M* = 64.04, SD = 5.93) and those who completed the pre-Human Library CCA only (*n* = 17, *M* = 59.11, SD = 8.23). No significant differences were detected between pre-Human Library CCB or CCA scores for participants who completed or not completed both pre and post-test surveys. This may indicate that completion of both pre and post-Human Library CCA had a moderately positive influence on pre-Human Library CAS scores. Therefore, participants who completed both pre and post-CCA started at a higher level of cultural awareness and sensitivity than those who completed the pre-Human Library CCA only. (See Table 2).

For participants who completed both pre and post Human Library surveys, significant differences in pre-Human Library CAS scores were observed between male and female (*p* = 0.005); and participants with or with or without previous work experiences in aged care, disability and health (*p* = 0.047). CAS, CCB and CAS scores between subgroups of participants based on their location or previous cultural learning/interaction were not significantly different.

### Qualitative analysis

Three participants consented to participating in the qualitative phase of the research. Two participants joined a focus group and one participated in a single interview. Audio recordings were transcribed verbatim by the lead researcher and de-identified replacing names with pseudonyms during the transcription process (29). Two main themes resulted from analysis of the qualitative data including: cultural competence prior to the Human Library and the influence of the Human Library on cultural competence.

#### Cultural competence prior to the Human Library

Participants felt they already had a level of cultural awareness and competence prior to the Human Library, influenced partly by personal experience, including the cultural environment they grew up in “the school I went to was very diverse” (Abbi), with one international student noting:

“I think in my culture people are still not very open to having touch, like they are not very used to hugging each other or like kissing or whatever… they keep the distance between people, but in Western culture, they are more open to having physical touch” (Belle).

Participants reflected on the influence media had on their assumptions about others with one saying: “The main assumptions I had was just the stuff I was seeing through movies and social media” (Abbi).

Participants noted that prior university-based cultural learning experiences facilitated improved awareness of, and sensitivity to, differences between cultures:

“I learnt a lot this year and last year at uni about certain cultures…” (Abbi).

“After this subject I think I kind of more understand and more open to different culture and yeah, and I will understand more um the thought of different culture” (Belle).

A capacity for culturally competent behavior was demonstrated prior to the Human Library by some:

“If you put it into action, um… you meet them and you get to talk with them, you will try to avoid kind of like, letting those stereotypes and assumptions affect your relationship with them” (Ash).

Whilst others reflected on their learning needs in this area:

“I suppose I have not properly worked out how to control the, you know sort of modify how I act to suit everyone’s different needs, cause obviously they will not all have the same views and values” (Abbi).

Despite reporting the positive impact of university activities on their levels of cultural competence, participants identified barriers to opportunities for intercultural interactions, including personal barriers such as confidence:

“I think I am introvert and not very good at opening up a topic and even in my same language I’m not very good at speaking very confidently or speaking very fluently” (Belle).

The impact of online learning due to coronavirus disease in 2019 (COVID-19) on opportunities to have personal intercultural interactions was noted by participants:

“At university, um maybe because I had only 1 day at uni and the rest of it was online via tutorials, so you did not really get the chance to connect with people” (Abbi).

“Especially in the pandemic situation, I do not have many chances to really see people or communicate with different people so yeah, it give me one more chance to” (Belle).

#### Influence of the Human Library on cultural competence

Participants viewed the Human Library as another opportunity to practice intercultural interactions and highlighted the importance of listening:

“I think it’s just about giving me more chance to practice or to face different people from different culture” (Belle).

“From listening alone, you can learn a lot about a person’s culture and life story” (Ash).

The Human Library encouraged new realizations and new concepts for participants with one participant remarking on an older human book’s use of technology:

“I did not take into consideration that we were in the 21st century and they may have like other family members who have taught them with the technology or maybe they have like a lot of exposure already throughout the years and they may be more experienced than us” (Ash).

Participants experienced moments of surprise when their unconscious assumptions were challenged As illustrated by a participant who gained insight that a mother could still be very actively involved in providing care for her son, even though he is an adult.

“My initial impression on this was that the mother has a very young son, less than 10 years old, but I did not expect him to be a full grown man” (Ash).

Another participant reflected that the story she heard helped her to consider that the skills of healthcare professionals does not guarantee acceptance from the client:

“I thought she must be very capable and very skilled in handling this issue but after her presentation, she mentioned her brother not willing to accept her help she can do nothing” (Belle).

The Human Library encouraged participant self-reflection and highlighted differences between people when human books, and their stories, did not meet expectations. Self-reflection helped students identify that their own values could affect others and that a person’s views and values can influence their behavior:

“Reflecting on how my values affect other people and understanding that other people have different views and values to me and then obviously that will affect how they behave as well and finding the mutual respectfulness balance” (Abbi).

The Human Library encouraged another participant to reflect on her own life situation and common humanity when reflecting on the experience of the human book:

“She has to take care of um a disabled family member which make me for um feel I’m so glad to um live in a relatively perfect um situation which I do not have to worry too much” (Belle).

Participants recognized how the pre and post-Human Library reflection activities, and completion of the CCA, helped them get more from the Human Library:

“I think the interview itself was amazing but I do not think I would have taken away as much if I did not do the reflective part…cause otherwise I would have attended, listened and moved on. I really liked that part, it really made you think when you do the survey” (Abbi).

The Human Library served as a reminder to participants to be aware of their own unconscious beliefs, values, and biases; and to continue towards developing culturally competent behavior. One participant felt that, although their cultural awareness and sensitivity did not necessarily change as a result of the Human Library, it reminded them to continue to reflect and inspired them to take action to promote culturally congruent healthcare practice:

“I think my willingness stayed the same, it maybe just reminded me to do more” (Ash).

When asked how they would do more, they responded:

“Maybe get an interpreter beforehand discuss what you are gonna talk about so that there’s no misinterpretations when the interpreter is translating. And also, if you are about to suggest like recommendations or an intervention plan to the client from a different culture, maybe do a bit of research beforehand to see if it aligns with their culture and their customs” (Ash).

Another participant reflected that they had never asked friends about their different cultures and that those conversations would be important moving forward into clinical placement:

“Having those conversations like we do not normally do would be a first step and it’s not a difficult step either” (Abbi).

Similarly, a participant identified that as a result of the Human Library; they would explore other opportunities to interact with people from different cultures:

“I think maybe join more different volunteers or explore more chances to get in touch with different culture” (Belle).

The same participant, when asked what they thought might change as a result of the Human Library, talked about the importance of considering people’s different backgrounds and perspectives, emphasizing that they learned not to judge others by their appearances:

“I will take more consideration about people—everyone has their life story, and we have to respect their story and we should not um give a first impression about their appearance or… um yeah we do not have… we should not judge the person about their appearance but we have to understand that they have different stories” (Belle).

## Discussion

The aim of this research was to examine the effect of participation in a Human Library on the development of occupational therapy (OT) students’ cultural awareness and sensitivity. The quantitative findings from the pre-post CCA (25) indicate that some students had higher levels of cultural awareness and sensitivity, and culturally competent behaviors than others prior to the Human Library. Factors influencing these baseline scores were gender and work experience (client layer).

The influence of gender on the baseline cultural awareness and sensitivity scores was noted with women having statistically significantly higher levels than men as measured by the CAS (25). Interestingly, this difference was not carried through to the post Human Library scores which may indicate that males’ levels of CAS improved more than females as a result of the Human Library.

The influence of previous work experience on cultural awareness and sensitivity was noted with statistically significantly higher levels recorded on the CAS (25) for participants with work experience in health, aged care or disability. Presumably some of these participants would have been exposed to people from different cultural backgrounds in their work allowing for opportunities to reflect on these interactions and potentially build awareness and sensitivity to cultural differences.

Eighty two percent of participants reported having daily to weekly intercultural interactions at work/study compared to 38 percent at home or during leisure time. This potentially reflects that the research was undertaken at a time of enforced COVID 19 restrictions which dramatically reduced opportunities for intercultural interactions for many participants, particularly within their personal lives. As one interview participant pointed out, the students in this cohort had 1 day of face-to-face learning since starting university in March 2020 followed by mostly online learning into 2021, during which the Human Library took place. As suggested in a recent report by the Tertiary Education Quality and Standards Agency in Australia (30) one of the main negative effects of an online university environment is student social isolation, lack of engagement with others, and reduced motivation. Qualitative findings support this in that students identified feeling isolated from peers, despite the online learning environment, citing lack of informal interactions and reduced access to shared social spaces.

Significant positive changes from pre to post Human Library CAS, CCB and CCA scores supported that the Human Library had a significant impact on increasing students’ cultural awareness and sensitivity, as well as self-perceived culturally competent behaviors. Even with participants who had previous work experience having significantly higher baseline CAS scores, the differences in CAS scores between those who had work experience and those who had not were not significant post Human Library. This suggests that students with less exposure to diverse populations through work might benefit more from this program. This reflects previous studies on the Human Library which noted how the activity highlights differences between people and serves to draw attention to discriminatory attitudes and stereotyping (9, 11, 13). Cultural competence can be seen as a behavioral construct that includes the ability to learn about people from different cultures, and adapt care to meet their needs (15). The Human Library helped some participants build on their prior levels of cultural awareness and sensitivity to begin considering how they could modify their behavior to provide culturally congruent services. Participants for example, discussed accessing resources to ensure their future therapy interventions aligns with cultural needs, having conversations with clients about their cultural preferences, and exploring more opportunities for further intercultural interactions to improve readiness for clinical placement.

Experiential learning methods, especially ones that target diversity awareness *and* behavior change can be instrumental in helping healthcare profession students become culturally congruent practitioners (31). Importantly the positive effect the Human Library had on students’ development of cultural competence in this current study supports findings of others that local experiential methods for developing cultural competence, such as the Human Library, are more financially and logistically viable than immersive experiences (32, 33). Remuneration for human books who wish to tell their stories or make a social impact, the hiring of facilities (if not available in kind), and catered refreshment breaks is much less expensive than immersive experiences, especially those involving international travel to live or study abroad (32, 33). Furthermore, as was needed for this study due to COVID-19 restrictions, the Human Library can be conducted online and still produce positive changes in cultural competence. This reflects previous studies exploring low-cost educational methods for the development of cultural competence (34–37). From an educators’ point of view a Human Library, whether in person or online, therefore provides the opportunity for a more accessible and equitable learning method for large cohorts of healthcare students.

Qualitative findings suggest that participants made gains in cultural awareness and sensitivity through the pre and post Human Library self-reflection activities with one participant noting that they would not have gained as much from the experience without the reflective component. Although the findings were based on three participants, the use of reflection as a transformative process and to develop deeper learning is commonly documented in the literature (38) with reflection enabling people to align their own values with their professional actions (39). In the context of culturally safe care this is an important aspect of the Human Library activities. Cultural safety builds on awareness and sensitivity and requires a continual process of reflection on practice with the health professional reflecting on their own cultural identity and privilege (4, 40, 41). Reflective tasks are therefore built into the educational element of the Human Library as mandatory submitted pass/fail tasks, but were not part of the research analysis. This was due to consideration of potential student/research bias with students knowing that the research analysis was conducted by academics associated with the program. As Cranton (42) notes, the importance of learners’ privacy needs to be respected and consideration given to how reading personal reflections could induce the learner to write what they think the educator wants to see. Reflection should therefore be a fundamental part of a Human Library experience from an educational and cultural safety perspective but future research will need to consider how to use this data without compromising disciplinary power (42).

### Limitations

The measurement tool used in this study, the CCA (25) measures cultural awareness and cultural sensitivity as one construct which potentially means that changes in cultural sensitivity as a result of the Human Library are not captured independently from cultural awareness in this study. The results of this study represent data collected from one cohort of undergraduate second-year students, from one university who had fewer opportunities to interact with people during the COVID pandemic. We therefore need to be cautious in interpreting the results. Although the results of this study supported the use of the Human Library together with reflection to enhance cultural competency development, we do not know how the effect of this pedagogy compares with other teaching methods. We aimed to collect data from a larger sample size, but the response rate was not high. A small sample size limited our ability to measure the influence of various factors that may contribute to one’s cultural competency. In future research, conducting randomized controlled studies to compare Human Library and teaching methods would provide further evidence for the effectiveness of using a Human Library to facilitate the development of cultural competency. Qualitative sampling did not result in participant numbers suitable for purposive sampling or a focus group. The opinions of the three participants could be biased and therefore limit their ability to explain quantitative data. A larger sample size would be needed to reduce this bias in future research. It is also difficult to generalise the findings to the wider health student population

## Conclusion

The findings of this small study suggested that gender and having experiences in working in the aged care, disability and health sector in a country with a diverse population have a positive influence on the person’s cultural awareness. However, the factors may not have association on a person’s cultural behavior. As recommended by Bezrukova et al. (31) teaching diversity and cultural competence is best accomplished using integrated, mandatory, cumulative and multi-modal approaches across a curriculum rather than stand-alone approaches. The Human Library, as a social experiential learning opportunity, can be considered for inclusion within a suite of learning methods for healthcare professional student curricula as a cost-effective, flexible teaching method for cultural competence. It offers a unique opportunity to listen to a person’s story and learn not only about the storyteller, but also about ones’ self. When offered early in the curriculum and in conjunction with other teaching methods including self-reflection activities, the Human Library can serve to facilitate student healthcare professionals to progress from cultural awareness and sensitivity towards the building of culturally competent behaviors and ultimately the provision of culturally congruent care.

## Data availability statement

The raw data supporting the conclusions of this article will be made available by the authors, without undue reservation.

## Ethics statement

The studies involving humans were approved by Monash University Human Research Ethics Committee. The studies were conducted in accordance with the local legislation and institutional requirements. The participants provided their written informed consent to participate in this study.

## Author contributions

EC, KP, and HH-V: conceptualization, writing-review and editing. EC and HH-V: quantitative data analysis. HH-V and KP: qualitative analysis. All authors contributed to the article and approved the submitted version.

## Conflict of interest

The authors declare that the research was conducted in the absence of any commercial or financial relationships that could be construed as a potential conflict of interest.

## Publisher’s note

All claims expressed in this article are solely those of the authors and do not necessarily represent those of their affiliated organizations, or those of the publisher, the editors and the reviewers. Any product that may be evaluated in this article, or claim that may be made by its manufacturer, is not guaranteed or endorsed by the publisher.
